# Melatonin-mediated dual regulation of physiological-biochemical traits and rhizosphere microbial communities alleviates drought stress in *Bougainvillea*

**DOI:** 10.1186/s12870-026-09179-1

**Published:** 2026-06-06

**Authors:** Lele Wang, Yefang Li, Wei Zhang, Yan Zhao, Yurong Lu, Jie Song, Wenling Guan

**Affiliations:** 1 College of Resources and Environment, Yunnan Agricultural University, Kunming, China; 2 College of Landscape and Horticulture, Yunnan Agricultural University, Kunming, China; 3 Flower Research Institute of Yunnan Academy of Agricultural Sciences, National Engineering Research Center for Ornamental Horticulture, Kunming, China; 4 College of Agronomy and Biotechnology, Yunnan Agricultural University, Kunming, China

**Keywords:** *Bougainvillea*, Melatonin, Drought stress, Rhizosphere microbiome

## Abstract

**Background:**

Drought conditions limit the distribution of *Bougainvillea* varieties, reduce their ornamental value, and increase maintenance costs. Although the rhizospheric melatonin-mediated mechanisms of plant drought resistance have been extensively documented. its role in the drought stress response of *Bougainvillea*, especially the regulatory effect on rhizosphere microorganisms, remains unclear. Using *Bougainvillea glabra* ‘Cypheri’ as plant material, this study employed a pot-based natural drought experiment combined with physiological indicator measurement, rhizosphere microbiome high-throughput sequencing, microbial network analysis, PICRUSt2 functional prediction, and redundancy analysis (RDA) to systematically investigate the regulatory mechanisms of melatonin on aboveground-belowground synergistic drought resistance.

**Results:**

Compared with the control group, drought stress significantly enhanced the activities of superoxide dismutase (SOD), peroxidase (POD), and catalase (CAT), as well as the proline (Pro) content in *Bougainvillea*, while increasing malondialdehyde (MDA) accumulation. Conversely, leaf relative water content (RWC) and chlorophyll (Chl) content were markedly reduced. Simultaneously, the rhizosphere microbial community balance was disrupted, bacterial network robustness diminished, and carbon metabolic pathways were suppressed. Exogenous melatonin application further significantly enhanced antioxidant enzyme activities and Pro accumulation under drought stress, reduced MDA content, and improved the physiological status of plants. Simultaneously, it promoted the enrichment of Proteobacteria, modulated the abundance balance of Ascomycota, enhanced microbial network stability, and optimized carbon, nitrogen, and energy metabolic pathways. Collectively, these results demonstrate that melatonin enhances drought tolerance in *Bougainvillea* by regulating the structure and network stability of rhizosphere microbial communities, synergistically improving aboveground drought resistance physiology, and mediating aboveground–belowground synergistic interactions.

**Conclusion:**

This study provides a theoretical foundation for the application of melatonin in drought-resistant cultivation practices for *Bougainvillea* species.

**Supplementary Information:**

The online version contains supplementary material available at 10.1186/s12870-026-09179-1.

## Introduction

*Bougainvillea*, a genus belonging to the family Nyctaginaceae, is widely recognized for its ornamental value and has also received increasing attention for its potential medicinal significance [1]. Previous studies have identified diverse bioactive compounds in *Bougainvillea* species and demonstrated their pharmacological activities, including antioxidant and anti-inflammatory effects [2, 3]. Meanwhile, Characterized by a diverse range of flower colors, an extended flowering duration, and ease of cultivation, *Bougainvillea* species find extensive application in landscape greening [4]. However, owing to challenges in regular maintenance, seasonal drought, and urban water scarcity, plants are often subjected to prolonged drought conditions in landscape environments. Although *Bougainvillea* exhibits moderate drought tolerance, its growth remains relatively sensitive to water availability. Prolonged water deficiency can lead to premature abscission of flowers and leaves, diminished ornamental value, and in severe cases, plant mortality [5].

Exogenous application of plant growth regulators has been widely demonstrated to alleviate the adverse effects of environmental stresses on plants [6–8]. Melatonin, an indole compound ubiquitously present in plants, is not only an important plant growth regulator but also a potent free radical scavenger and antioxidant, playing a critical role in plant responses to both biotic and abiotic stresses [9, 10]. In recent years, melatonin has shown considerable potential in mitigating drought stress [11]. Previous studies have demonstrated that exogenous melatonin can significantly enhance plant drought tolerance by increasing antioxidant enzyme activities, maintaining cellular osmotic balance, improving photosynthetic efficiency, and regulating physiological, biochemical, and metabolic processes [12–15]. For example, Mehralian et al. [16] found that melatonin alleviated drought stress in *Dracocephalum kotschyi* Boiss. by modulating physiological, biochemical, and metabolic status. Eghlima et al. [11] further reported that melatonin markedly enhanced drought tolerance in *Thymus vulgaris* L. by activating enzymatic scavenging systems, reducing oxidative damage, and regulating cellular osmotic balance. Although the drought-alleviating effects of melatonin have been confirmed in various crops and herbaceous plants [17–19], studies on its application in woody plants, particularly woody ornamental species, remain relatively limited, and the underlying regulatory mechanisms are still poorly understood. Therefore, elucidating the mechanisms by which exogenous melatonin mitigates drought stress in *Bougainvillea* is of great practical significance for improving its drought adaptability, expanding its cultivation range, and broadening the application of melatonin in drought regulation of woody ornamental plants.

Current studies have mainly focused on the direct effects of melatonin on plant physiological metabolism and molecular regulation, whereas comparatively less attention has been paid to its indirect role in enhancing plant drought resistance through modulation of rhizosphere microbial communities [20]. The rhizosphere is an important microecological interface connecting plant roots and the soil environment, harboring diverse microbial communities that are deeply involved in nutrient cycling, material transformation, host plant growth and development, pathogen suppression, and stress adaptation [21, 22]. As one of the most typical and destructive abiotic stresses, drought can alter soil moisture conditions and nutrient availability, thereby significantly affecting the diversity, composition, and stability of rhizosphere microbial communities [23]. Recent studies suggest that melatonin not only directly regulates plant stress physiology but may also enhance drought tolerance by reshaping the structure and function of rhizosphere microbial communities and creating a more favorable rhizosphere microenvironment [24, 25]. Melatonin has been reported to promote plant growth and nutrient utilization in peanut [26], reprogram rhizosphere microbial communities under abiotic stress [27], and significantly alter the functional characteristics of bacterial communities under drought conditions in barley [20]. In addition, Zhang et al. [28] demonstrated that melatonin could induce plants to recruit potentially beneficial microorganisms, thereby enhancing drought tolerance and post-stress recovery capacity. However, the mechanisms by which melatonin regulates the dynamic changes of rhizosphere microbial communities under drought stress remain insufficiently understood. In particular, further investigation is needed regarding the identification of potentially beneficial microorganisms, the regulatory effects of melatonin on microbial network stability, and the underlying mechanisms involved.

This study systematically investigated the effects of exogenous melatonin application on the growth and physiological characteristics of *Bougainvillea* under drought stress, while also analyzing its regulatory impact on the structure, functional metabolism, and network stability of the rhizosphere microbial community. Based on the relevant research foundation and background, we propose the following hypotheses: (1) Melatonin alleviates drought-induced damage in *Bougainvillea* by activating the plant’s antioxidant defense system; (2) Melatonin enhances the stability of the microbial network by modulating interactions within the rhizosphere microbiome, thereby helping the plant counteract drought-induced microecological disturbances; (3) Melatonin improves the plant’s drought resistance by regulating the structure of the rhizosphere soil microbial community, which synergistically enhances the above-ground physiological drought tolerance, achieving an above-ground-belowground synergistic interaction. This study aims to systematically elucidate the comprehensive mechanisms by which melatonin enhances drought resistance in *Bougainvillea* from both plant physiological metabolism and rhizosphere microbial community perspectives. The findings are expected to provide a scientific theoretical basis and research direction for the rational application of melatonin in drought-resistant cultivation of *Bougainvillea*, while also offering new insights and technical references for drought regulation in woody ornamental plants.

## Materials and methods

### Experimental materials

In this study, *Bougainvillea glabra* ‘Cypheri’ cultivated in the greenhouse of the College of Landscape and Horticulture, Yunnan Agricultural University(25°08’N, 102°76’E), was employed as the experimental material. Three-month-old cutting seedlings of *Bougainvillea glabra* ‘Cypheri’ were transplanted into plastic pots (17.5 cm in diameter, 14.5 cm in height) contained 2 kg of the same homogenized substrate (red soil: coconut husk: coco peat: perlite = 3:2:3:2). The substrate had a pH of 6.0, with the following nutrient contents: total nitrogen 0.84 g/kg, total phosphorus 1.7 g/kg, total potassium 6.35 g/kg, hydrolyzable nitrogen 76 mg/kg, available potassium 642 mg/kg, and available phosphorus 55 mg/kg. The potted plants were cultivated in a greenhouse under controlled conditions: a temperature of 25 ± 3℃, relative humidity between 60% and 70%, and exposure to natural sunlight. After two months of cultivation under these conditions, the plants were subjected to experimental treatments.

### Experimental treatment

Three treatments were established: Control (CK), Drought (DR), and Melatonin + Drought (MT). The melatonin concentration (200 µM) was selected primarily based on previous literature reporting effective doses for drought stress alleviation in plants [16, 20], and preliminary observations indicated that this dose was appropriate for *Bougainvillea* under the present experimental conditions. Prior to drought induction, plants in the MT treatment were irrigated at the root zone with 300 mL of melatonin solution per plant once every two days for a total of five applications. Throughout the experimental period, both the CK and DR groups were administered an identical volume of distilled water during each irrigation event. Following the melatonin pretreatment, the DR and MT treatments were subjected to natural drought stress until the soil water content declined to 5–10%, with the drought period lasting for 28 days. The CK treatment received regular watering throughout the experiment. Each treatment group comprised four biological replicates, with each replicate containing four seedlings. With the onset of drought designated as day 0, mature leaves from the same middle growth position of the plants were collected at day 0, day 14, and day 28. One portion of the leaves was used for the determination of leaf water content, while the other portion was immediately flash-frozen in liquid nitrogen and subsequently stored in a -80℃ freezer for further analysis. On day 28, entire plants were carefully harvested from their pots. Following gentle shaking to dislodge loosely attached soil, the rhizosphere soil was meticulously collected from the roots by brushing. The gathered soil was subsequently passed through a 2-mm sieve to remove any root fragments and debris. The rhizosphere soil from four plants within each replicate was combined into one composite soil sample, for a total of four replicates. For microbial community analysis, soil samples were placed in sterile tubes, immediately snap-frozen in liquid nitrogen, transferred to the laboratory, and stored at − 80 °C until further processing.

### Measurement of growth physiological parameters

The leaf relative water content (RWC) was assessed using the following procedure: For each treatment, four fresh leaves were randomly sampled and weighed to determine fresh weight(FW). After 24-hour immersion in distilled water, the saturated fresh weight(SFW) was measured. The leaves were subsequently oven-dried to constant weight and their dry weight(DW) was recorded.

The RWC was calculated using the formula: RWC(%)=(FW-DW)/(SFW-DW)×100.

The relative chlorophyll content of leaves from plants under different treatments was measured using a SPAD-502Plus chlorophyll meter. For each treatment at each sampling time, four plants were randomly selected, and three mature functional leaves from different positions on each plant were measured. The average value of readings taken from the upper, middle, and lower sections of each leaf was calculated.

Determination of Physiological Parameters: The activity of superoxide dismutase (SOD) was determined using the nitroblue tetrazolium (NBT) photoreduction method; The activity of peroxidase (POD) was measured by the guaiacol colorimetric method; The activity of catalase (CAT) was assessed via the ultraviolet absorption method; The content of malondialdehyde (MDA) was determined using the thiobarbituric acid (TBA) method [29]. The proline content was quantified with a commercial kit produced by Beijing Hezi Biotechnology Co., Ltd. Detailed methodologies are provided in the appendix.

### Determination of soil microorganisms

Microbial Community Profiling and Sequencing Methodology. Soil microbiome sequencing services were provided by Shanghai Majorbio Technology Co., Ltd. Total genomic DNA was isolated from soil samples with the E.Z.N.A.^®^ Soil DNA Kit (Omega Bio-tek, Norcross, GA, USA). For bacterial community analysis, the V3–V4 hypervariable regions of the 16 S rRNA gene were amplified with primer pair 338 F (5′-ACTCCTACGGGAGGCAGCAG-3′) and 806R (5′-GGACTACHVGGGTWTCTAAT-3′). Fungal communities were characterized by amplifying the ITS region using primers ITS1F (5′-CTTGGTCATTTAGAGGAAGTAA-3′) and ITS2R (5′-GCTGCGTTCTTCATCGATGC-3′). All amplified libraries were sequenced on the Illumina NextSeq 6000 platform under 2 × 250 bp paired-end configuration.

### Microbial network analysis

To evaluate the effects of melatonin on microbial network structure under drought conditions, co-occurrence networks of fungi and bacteria were constructed separately. To minimize the influence of rare amplicon sequence variants (ASVs) across treatments, ASVs with a relative abundance of < 0.01% were removed [30]. To avoid spurious correlations caused by zero values, taxa that were present in more than 75% of the samples within each treatment were retained [31]. Microbial co-occurrence networks were developed using Pearson correlation measures, retaining interactions with a coefficient (r) greater than 0.7 and a significance level of *p* < 0.01 following false discovery rate (FDR) adjustment. Key topological indices—such as node and edge counts, proportions of positive and negative correlations, average degree, mean path length, network diameter, density, and clustering coefficient—were computed with the “igraph” package. The resulting correlation matrices were imported into Gephi software, and network visualization was performed using the Fruchterman–Reingold layout algorithm [32]. Network robustness was assessed using a graph-based approach by randomly removing 50% of the taxa (with Zi > 2.0 and Pi ≤ 0.62) from each network and recalculating robustness [33, 34]. Network vulnerability was determined based on the maximum node vulnerability within each network [35, 36].

### Microbial network Zi-Pi analysis

Based on the filtered ASV abundance data (retaining ASVs with an occurrence frequency > 3/4 and a total relative abundance > 0.00001), Pearson correlation coefficients and their corresponding significance (p-values) between ASVs were calculated. Significant correlations satisfying|r|≥0.6 and *p* < 0.05 were selected to construct an undirected co-occurrence network. The fast-greedy algorithm was employed to identify network modules [37]. The ecological roles of nodes within the network were quantitatively analyzed using the Zi-Pi index:

- Zi (within-module connectivity) =(kwithin−k̄module)/SD(kmodule), where kwithin is the number of connections a node has within its own module, k̄module is the average kwithin of all nodes in that module, and SD(kmodule) is the standard deviation of kwithin within the module. This metric reflects the strength of a node's connectivity within its own module.
- Pi (among-module connectivity)=kbetween/ktotal, where kbetween is the number of connections a node has to nodes in other modules, and ktotal is the total number of connections of the node. This metric reflects the node's capacity to connect different modules.

Based on the classical thresholds (Zi = 2.5, Pi = 0.62), nodes were classified into four ecological roles: Peripheral node (Zi ≤ 2.5 and Pi ≤ 0.62), Module hub (Zi > 2.5 and Pi ≤ 0.62), Connector (Zi ≤ 2.5 and Pi > 0.62), and Network hub (Zi > 2.5 and Pi > 0.62). Zi-Pi scatter plots and box plots of Zi and Pi indices were generated, and the Kruskal-Wallis test was employed to analyze differences in node connectivity among different treatment groups.

### Data analysis

The physiological and biochemical data were analyzed using IBM SPSS Statistics 26(IBM, Inc., Armonk, NY, USA). All variables were subjected to one-way ANOVA, the Duncan’s test was further applied, with 𝑝<0.05 considered statistically significant. Redundancy analysis (RDA) was performed to investigate the explanatory power and significance of rhizosphere microbial features in relation to the variation in physiological indices of *Bougainvillea*; and Spearman rank correlation analysis was combined to visualize the association patterns between physiological indicators and microbial characteristics All graphs were generated using R Studio 2025 and GraphPad Prism 8.

## Results

### Effects of melatonin on plant growth and physiological characteristics under drought stress

**Fig. 1:**
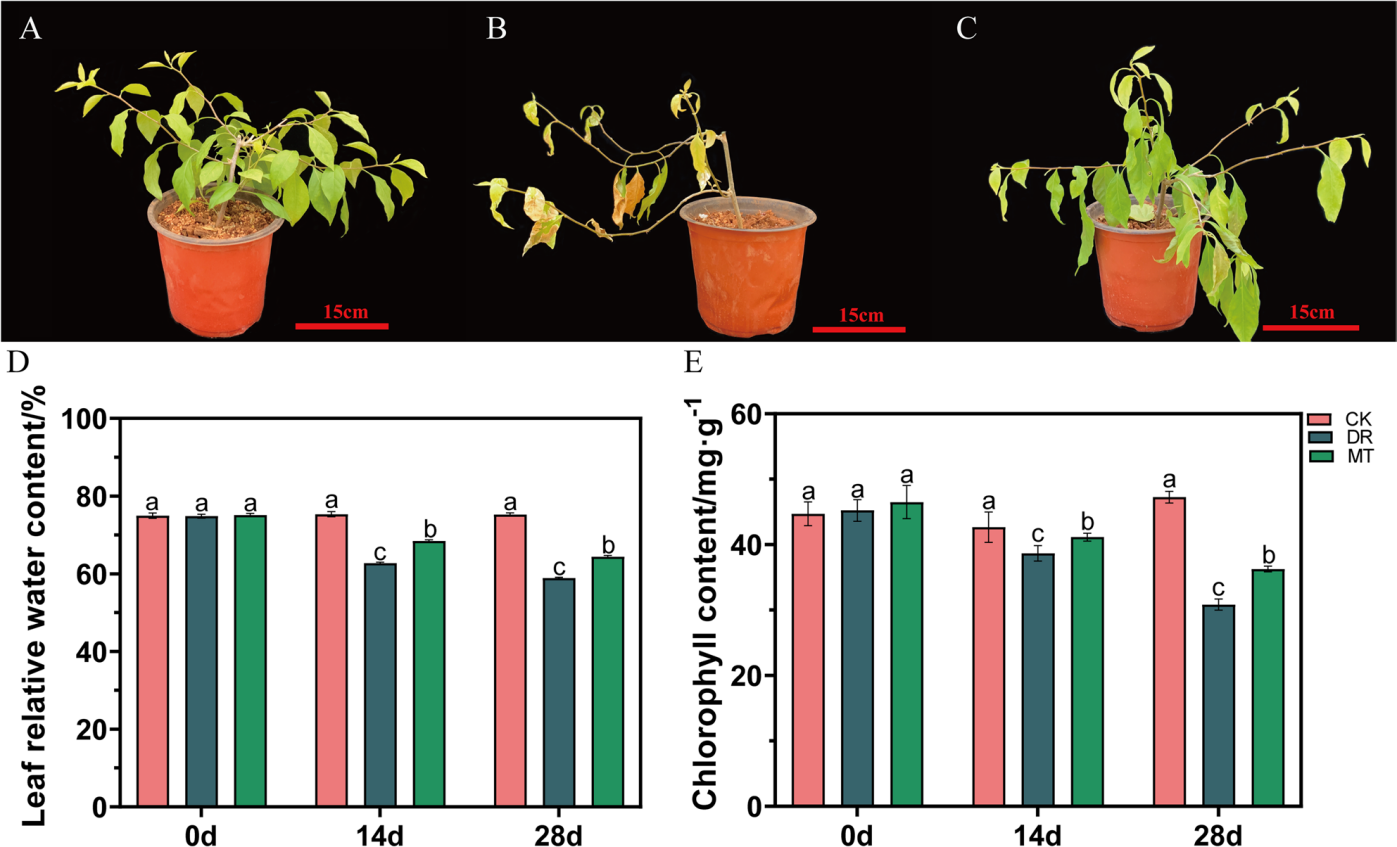
The effects of exogenous melatonin on the growth and physiological indicators of *Bougainvillea* under drought stress. (**A**) The morphological characteristics of plants under the CK treatment; (**B**) The morphological characteristics of plants under the DR treatment; (**C**) The morphological characteristics of plants under the MT treatment; (**D**) Influence of Exogenous Melatonin on Foliar Water Status in Drought-Stressed *Bougainvillea*; (**E**) Influence of Exogenous Melatonin on Chlorophyll Content in Drought-Stressed *Bougainvillea*. Different lowercase letters denote statistically significant differences among treatments at *P* < 0.05. CK: Control group; DR: Drought stress; MT: Melatonin treatment under drought stress

Drought stress significantly inhibited plant growth. Compared with the CK group (Fig. 1A), both DR and MT plants exhibited varying degrees of injury. After 28 days of natural drought, DR plants showed severe wilting, leaf yellowing and curling, and partial desiccation and abscission of branches and leaves (Fig. 1B). In contrast, drought-induced damage in MT plants was significantly alleviated, exhibiting only mild leaf drooping, and their overall growth status was markedly superior to that of the DR plants (Fig. 1C). At the initial stage of treatment (0 days), there was no significant difference in leaf RWC among the three groups, all maintaining a relatively high level of approximately 75%. As the drought duration extended, the RWC of DR leaves continued to decline, dropping to about 62.72% at 14 days, which was 16.75% lower than that of CK. By 28 days, it further decreased to approximately 58.94%, representing a 21.7% reduction compared to the CK group. In contrast, the RWC of MT plants was maintained at about 68.45% and 64.39% at 14 and 28 days, respectively, which was 9.14% and 9.25% higher than that of DR at the corresponding time points. This indicates that MT effectively alleviated drought-induced leaf water loss (Fig. 1D). Simultaneously, as the drought duration extended, the chlorophyll content in plant leaves gradually decreased. The chlorophyll content in the DR group continued to decline, showing a reduction of 9.31% compared to CK at 14 days and a more pronounced decrease of 34.75% at 28 days. In contrast, the chlorophyll content in the MT group was maintained at approximately 41.15 mg·g⁻¹ and 36.27 mg·g⁻¹ at 14 and 28 days, respectively. These values were 6.39% and 17.65% higher than those in the DR group at the corresponding time points, indicating that melatonin application significantly mitigated drought-induced chlorophyll degradation(Fig. 1E).

**Fig. 2:**
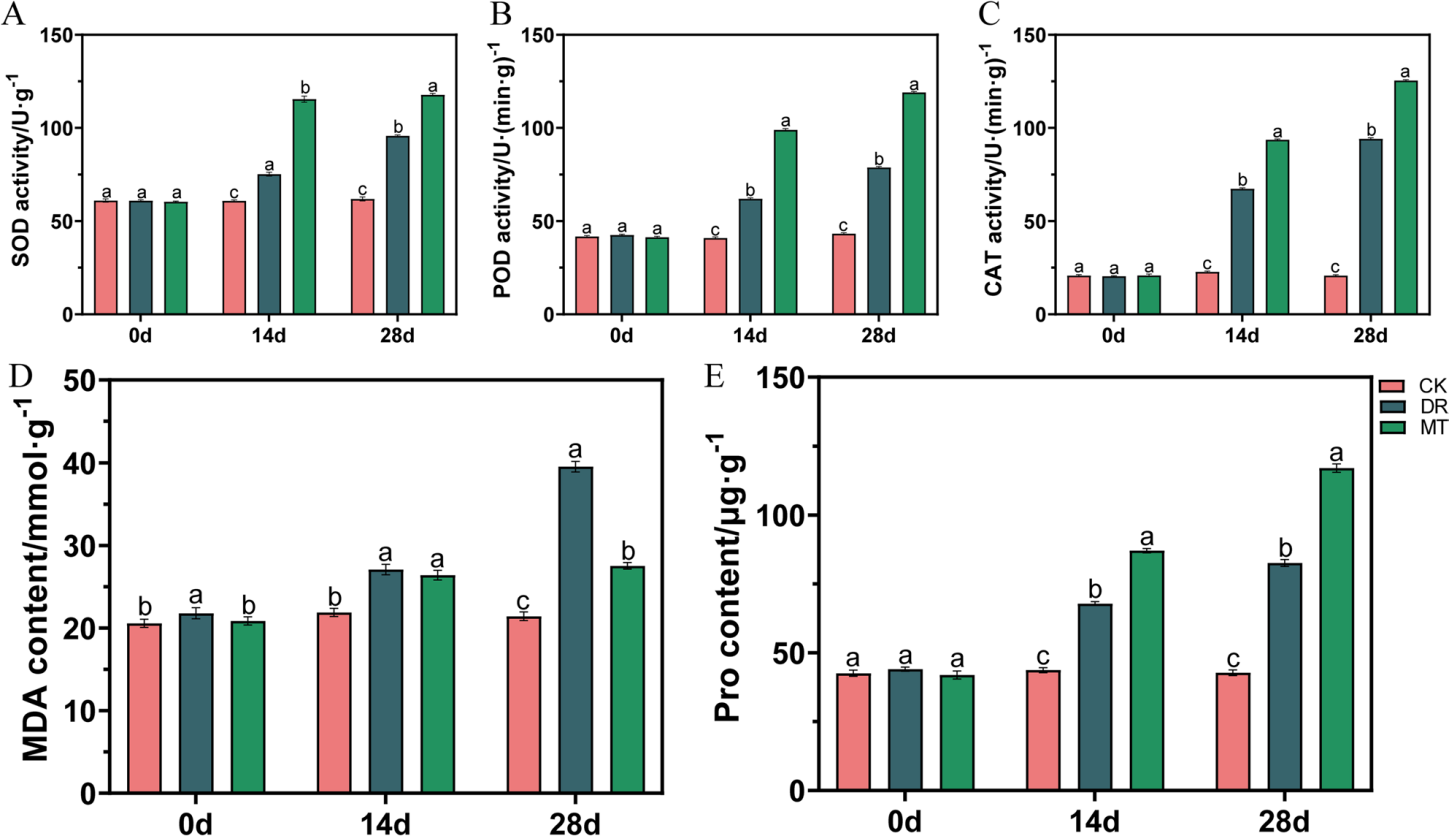
Melatonin-mediated changes in physiological parameters of *Bougainvillea* under drought stress. **A**.SOD activitiy; **B**.POD activitiy; **C**.CAT activitiy; **D**. MDAcontent; **E**. Pro content. Different lowercase letters denote statistically significant differences among treatments at *P* < 0.05. CK: Control group; DR: Drought stress; MT: Melatonin treatment under drought stress

As shown in Fig. 2, with the prolongation of drought stress duration, both the DR and MT treatments showed a significant increasing trend in the activities of SOD, POD, and CAT, with their activities at all time points being significantly higher than those of CK. Regarding SOD activity, the DR treatment significantly induced an increase in plant SOD activity at both 14 d and 28 d. The SOD activity in bougainvillea leaves under the MT treatment was 53.57% and 22.99% higher than that under the DR treatment at 14 d and 28 d, respectively (Fig. 2A), indicating that melatonin can further enhance SOD activity, thereby facilitating the scavenging of reactive oxygen species generated under drought stress. With respect to POD activity, drought stress significantly enhanced the POD activity in bougainvillea leaves regardless of melatonin application. Notably, the POD activity in the MT group was significantly higher than that in the DR group at both 14 d and 28 d, with increases of 59.65% and 51.13%, respectively (Fig. 2B). This indicates that melatonin effectively induces an increase in POD activity, thereby strengthening the plant’s antioxidant defense capacity. The trend in CAT activity was consistent with those of SOD and POD. As the drought duration increased, the CAT activity in all treatments increased progressively. Moreover, the CAT activity in the MT group was significantly higher than that in both the DR and CK groups at all drought time points (Fig. 2C), further confirming the activating effect of melatonin on the plant’s antioxidant enzyme system under drought stress.

With the prolongation of drought duration, the MDA content in the DR group continued to increase, showing a significant rise of approximately 23.76% compared to CK at 14 d and reaching its peak at 28 d with an increase of about 84.51% relative to CK. In contrast, the MDA content in the MT group was significantly lower than that in the DR group at the corresponding time points, exhibiting reductions of approximately 2.51% at 14 d and 30.3% at 28 d (Fig. 2D). These results indicate that melatonin effectively suppressed drought-induced membrane lipid peroxidation and alleviated cellular membrane damage. Drought stress significantly promoted the accumulation of proline, with a markedly greater accumulation amplitude observed in the MT group compared to the DR group. At 14 d, the proline content in the MT group was approximately 38.51% higher than that in the DR group. By 28 d, the proline content in the MT group reached its peak, showing an increase of about 41.65% compared to the DR group (Fig. 2E). These results indicate that melatonin enhances the plant’s osmotic adjustment capacity and maintains cellular water balance by promoting the synthesis and accumulation of proline.

### Influence of exogenous melatonin on the structure and diversity of the rhizosphere microbiome in drought-affected *Bougainvillea*

**Fig. 3:**
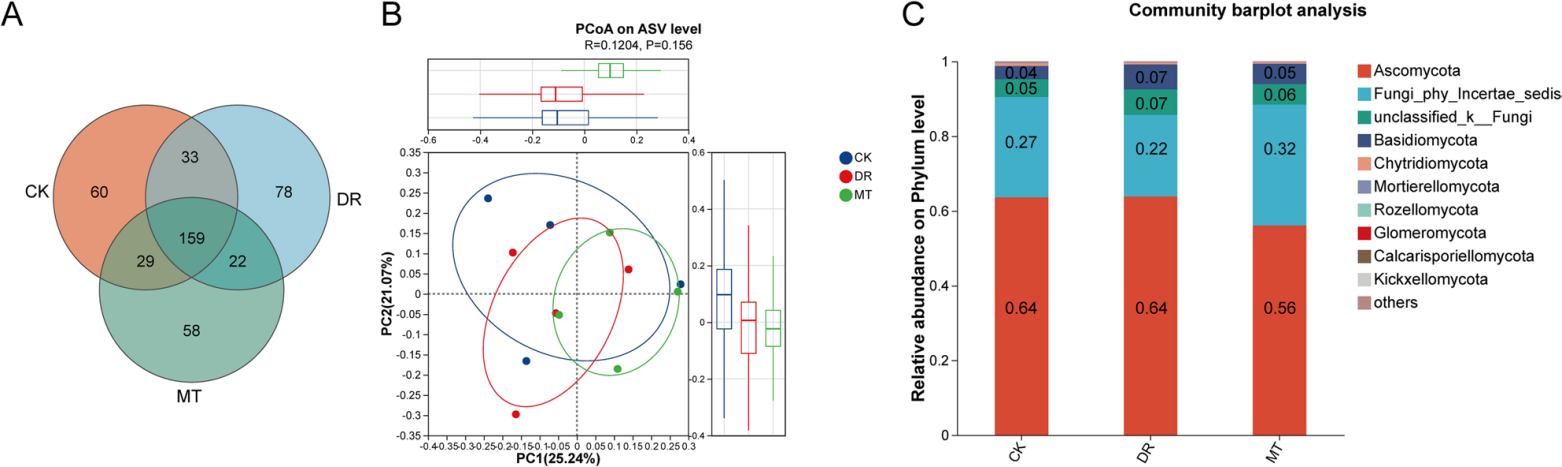
Influence of exogenous melatonin on the structure and diversity of the rhizosphere soil fungal communities in drought-affected *Bougainvillea*. **A**. Venn diagram; **B**.PCOA; **C**. The relative abundance of fungi at the phylum level. CK: Control group; DR: Drought stress; MT: Melatonin treatment under drought stress

**Fig. 4:**
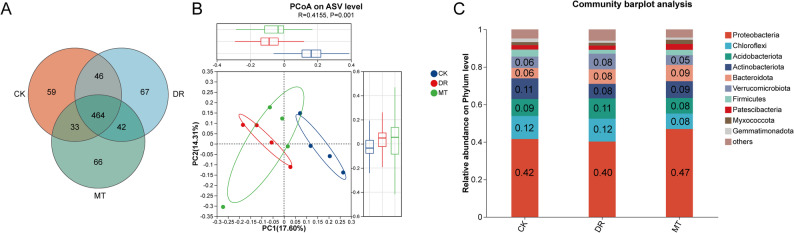
Influence of exogenous melatonin on the structure and diversity of the rhizosphere soil bacterial communities in drought-affected *Bougainvillea*. **A**. Venn diagram; **B**.PCOA; **C**. The relative abundance of fungi at the phylum level. CK: Control group; DR: Drought stress; MT: Melatonin treatment under drought stress

High-throughput sequencing of 16 S rDNA and ITS from 12 soil samples yielded a total of 439 fungal ASVs and 777 bacterial ASVs. In the fungal community, 159 core ASVs were shared among the three treatment groups (Fig. 3A), indicating that while drought and melatonin treatments altered the fungal community composition, a portion of the core taxa was retained. In the bacterial community, the number of shared core ASVs reached 464 (Fig. 4A), suggesting that the bacterial core taxa were highly conserved across different treatments, with differences primarily observed at the level of unique ASVs.

The α-diversity analysis of the rhizosphere soil fungal and bacterial communities in *Bougainvillea* indicated that neither DR nor MT treatments had a significant impact on the α-diversity indices of fungi or bacteria (*P* > 0.05). At the fungal community level, compared to the CK group, the DR group showed an increase in the Ace, Chao, and Shannon indices alongside a decrease in the Simpson index, reflecting a trend toward higher species richness and diversity. In contrast, the MT group exhibited a reduction in the fungal Ace, Chao, and Shannon indices and a slight recovery in the Simpson index compared to the DR group, with all metrics tending to revert toward the levels observed in the CK group, although the inter-group differences did not reach statistical significance (Fig. S1). The trend of change in bacterial community α-diversity was highly consistent with that observed in fungi (Fig. S2).

Principal coordinate analysis (PCoA) based on ASV-level data revealed that the differences in fungal community structure among the three treatments were not significant (*R* = 0.1204, *P* = 0.358); PC1 and PC2 accounted for 25.24% and 21.07% of the total variation, respectively (Fig. 3B). PCoA of the bacterial community indicated significant structural differences among the treatments (*R* = 0.4155, *P* = 0.001). PC1 and PC2 explained 17.60% and 14.31% of the total variation, respectively (Fig. 4B). Specifically, the bacterial community of the CK treatment was significantly separated from those of the DR and MT treatments, while the sample points of the DR and MT treatments showed no clear separation, indicating that drought stress significantly altered the bacterial community structure.

Among the top ten fungal taxa, Ascomycota was the dominant phylum with the highest relative abundance (Fig. 3C). The relative abundances of Ascomycota in the CK, DR, and MT groups were 63.67%, 63.88%, and 56.12%, respectively. The MT treatment significantly reduced its abundance, showing decreases of 13.45% and 13.83% compared to CK and DR, respectively.

In the bacterial community, Proteobacteria was the absolute dominant phylum (Fig. 4C). The MT treatment significantly increased the abundance of Proteobacteria, with increases of 11.34% and 14.32% compared to CK and DR, respectively.

### Network analysis of the rhizosphere soil microbial community of *Bougainvillea* under drought stress induced by exogenous melatonin

**Fig. 5:**
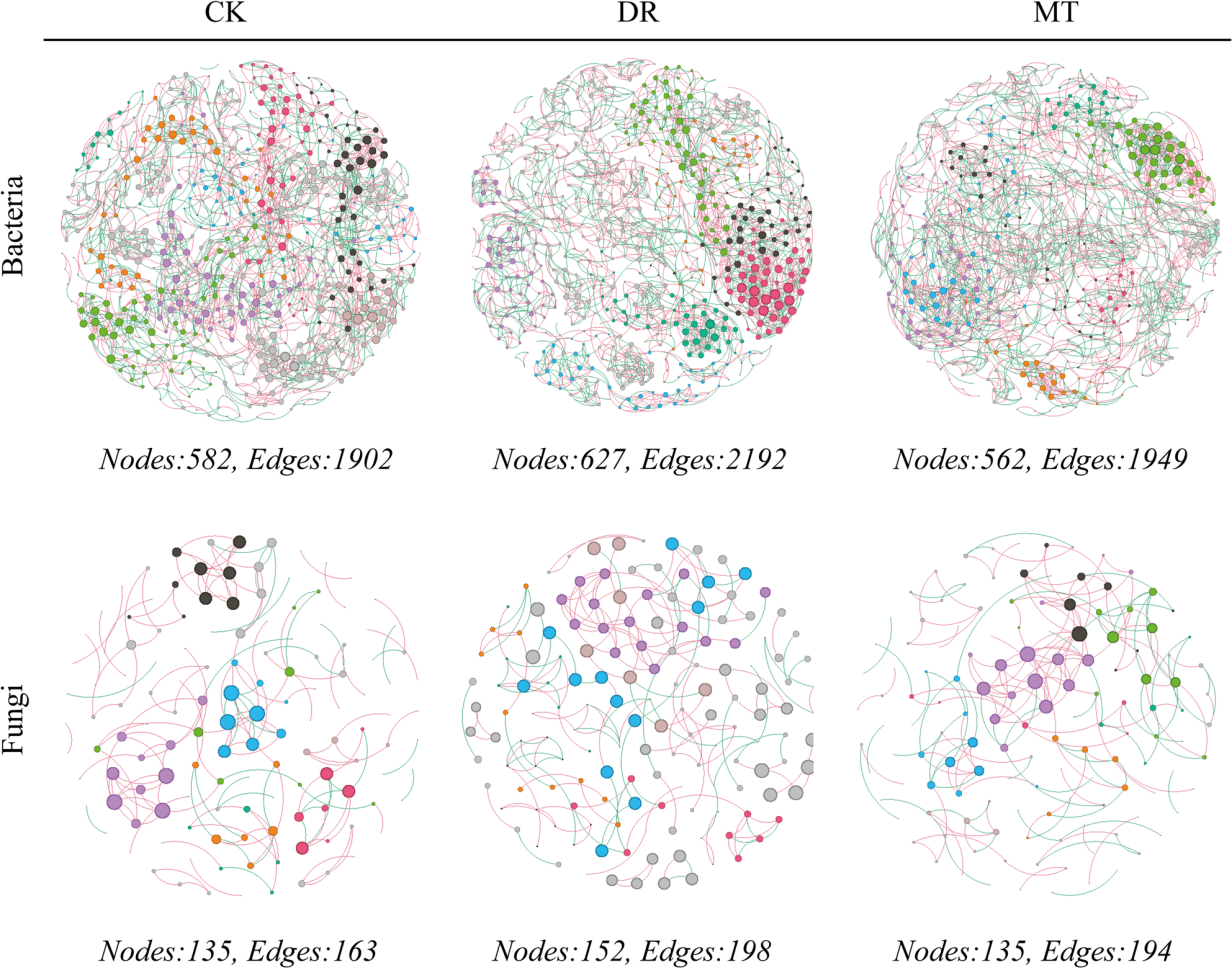
Network analysis of rhizosphere soil bacteria and fungi under different treatments. CK: Control group; DR: Drought stress; MT: Melatonin treatment under drought stress

To elucidate the regulatory effect of melatonin on the interactions within the rhizosphere soil microbial community of *Bougainvillea* under drought stress, this study constructed co-occurrence networks of bacteria and fungi and analyzed their topological properties. As shown in the visualized network (Fig. 5), both DR and MT treatments exerted noticeable effects on the number of nodes and edges in the bacterial and fungal networks. The fungal network under DR exhibited the highest number of nodes (152) and edges (198) among the three groups, surpassing those of CK (135 nodes and 163 edges). In contrast, the MT treatment showed no difference in the number of nodes compared to CK (135 nodes), but the number of edges (194) was slightly higher than that of CK, indicating that MT treatment enhanced the connectivity among fungal species. The bacterial network exhibited a trend consistent with that of the fungal network: compared to CK, the MT treatment showed a reduction in the number of nodes but a slight increase in the number of edges, indicating enhanced connectivity among bacterial species under MT treatment.

Compared to CK, both DR and MT significantly altered the topological parameters of the fungal network (Table 1). Under DR treatment, the fungal network exhibited increases in positive links, negative links, average degree, average path length, and network diameter, indicating a substantial enhancement in network complexity and connectivity under drought stress. Although the network density, clustering coefficient, modularity, and number of communities in the DR treatment were slightly lower than those in CK, the overall structure still reflected a trend toward greater complexity. In the MT fungal network, positive links, negative links, average degree, and average path length were all higher than those in CK, demonstrating that MT treatment also enhanced the complexity and connectivity of the fungal network. The changes in topological parameters of the bacterial network followed a trend highly consistent with that of the fungal network. The bacterial network under DR treatment exhibited higher positive links, negative links, average degree, and average path length compared to CK, indicating that drought stress markedly enhanced the strength of interactions among bacterial species and significantly increased network complexity. In the MT bacterial network, positive links, negative links, and average degree were all higher than those in CK, demonstrating that MT treatment also enhanced the complexity and connectivity of the bacterial network and alleviated the excessive disturbance caused by drought.

**Table 1:** Effects of exogenous melatonin on the topological features of fungal and bacterial networks in the rhizosphere soil of *Bougainvillea* under drought stress

Network indexs	Fungal community				Bacterial community		
	CK	DR	MT		CK	DR	MT
Positive links	123	132	126		997	1122	1034
Negative links	40	66	68		905	1070	915
Average degree	2.41	2.61	2.87		6.51	6.99	6.94
Average path lenth	2.85	4.05	2.74		12.48	12.42	11.30
Network diameter	7.96	13.92	8.94		30.77	33.73	31.77
Network density	0.018	0.017	0.021		0.011	0.011	0.012
Clustering coefficient	0.63	0.59	0.56		0.59	0.59	0.60
Modularity	0.904	0.896	0.842		0.864	0.854	0.859
Number of communities	33	30	26		21	18	26

### Effects of exogenous melatonin on the stability of rhizosphere soil microbial community networks in *Bougainvillea* under drought stress

**Fig. 6:**
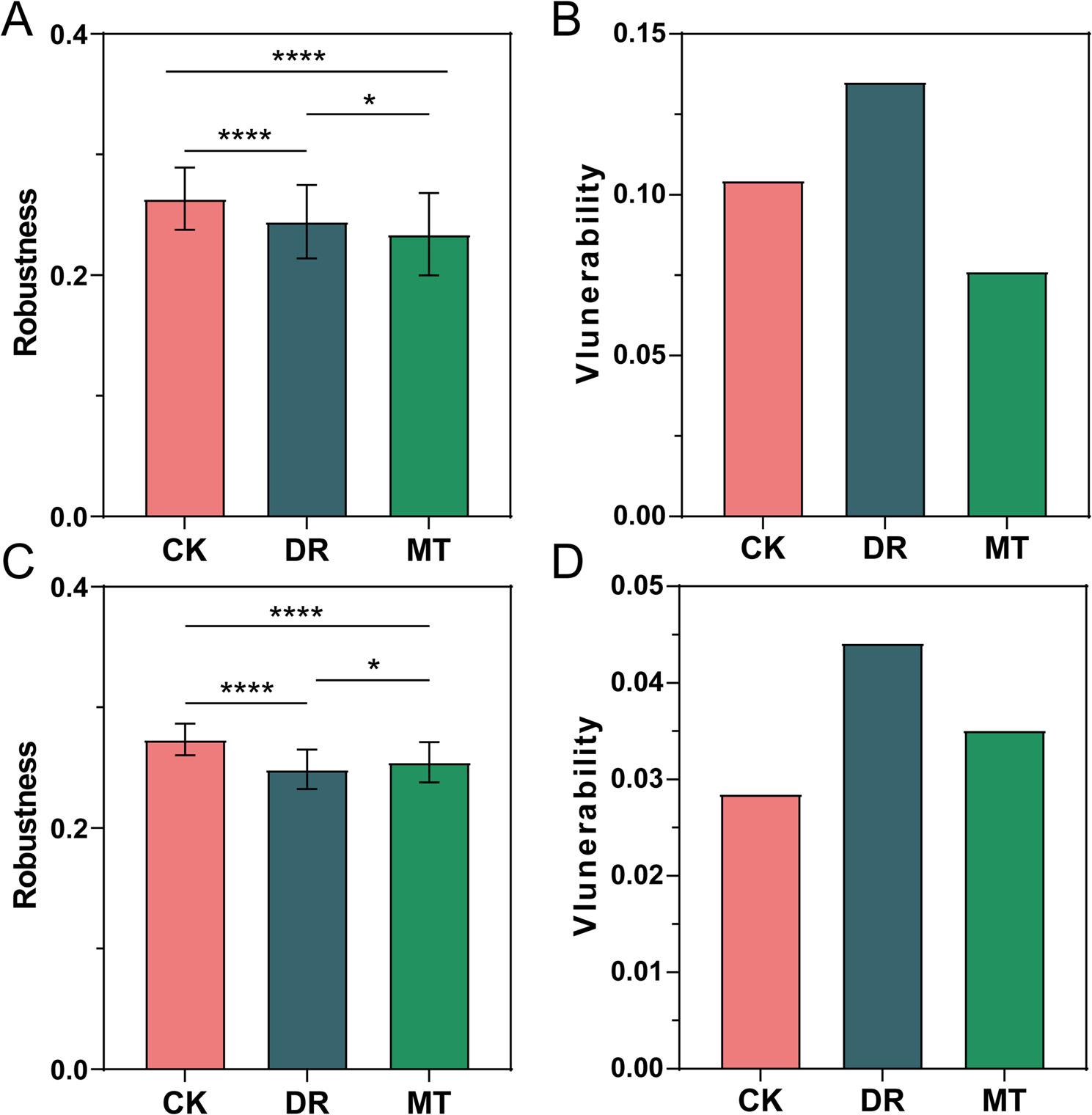
Effects of exogenous melatonin on the stability of rhizosphere microbial community networks of *Bougainvillea* under drought stress (**A**) Robustness of fungal networks; (**B**) Vulnerability of fungal network **C**. Robustness of bacterial network **D**. Vulnerability of bacterial networks

To evaluate the effects of drought stress and melatonin treatment on the stability of the rhizosphere microbial network in *Bougainvillea*, this study quantified the analysis using two indicators: robustness and vulnerability. In the fungal network, the robustness under both DR and MT treatments was significantly lower than that of CK (*P* < 0.0001), with MT exhibiting significantly lower robustness than DR (Fig. 6A). The trend in vulnerability was opposite to that of robustness, the fungal network vulnerability in the DR group was significantly higher than that in both CK and MT groups, while MT showed the lowest vulnerability (Fig. 6B). In the bacterial network, the trend of stability changes was similar to that observed in the fungal network, albeit with some nuanced differences. The robustness of bacterial networks under both DR and MT treatments was significantly lower than that of CK (*P* < 0.0001), and the robustness under DR was significantly lower than that under MT (Fig. 6C). The vulnerability of bacterial networks in both DR and MT groups was higher than in CK, with DR exhibiting the highest vulnerability, while the vulnerability under MT was lower than under DR (Fig. 6D). These results indicate that drought stress reduced the resistance of both fungal and bacterial networks to disturbances, whereas melatonin alleviated the vulnerability and enhanced the stability of these microbial networks under drought conditions.

### Zi-Pi analysis of microbial networks

**Fig. 7:**
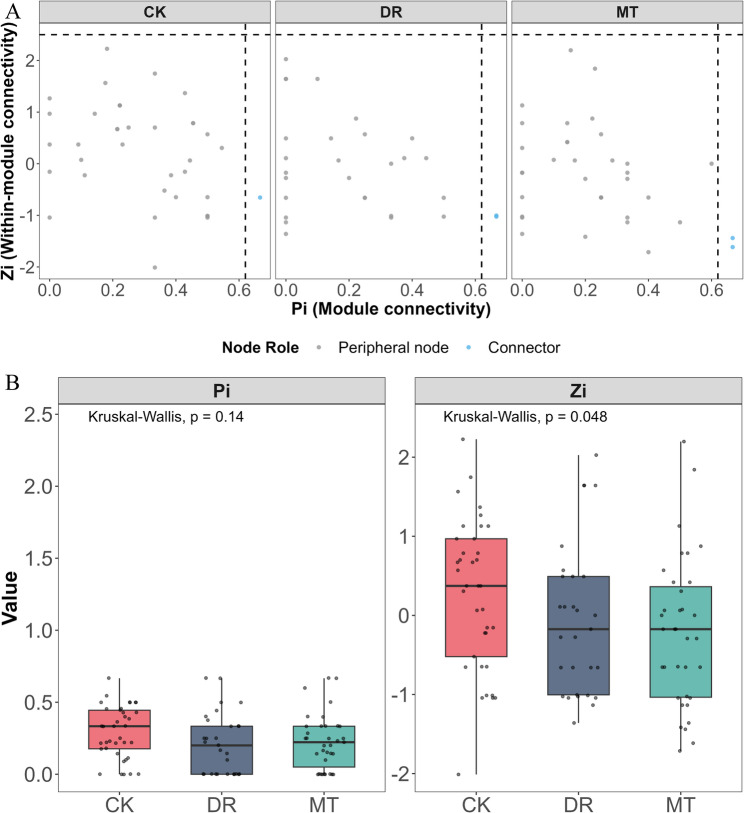
Zi-Pi analysis of fungal network nodes in the rhizosphere soil of *Bougainvillea*. (**A**) Scatter plot of node roles based on within-module connectivity (Zi) and between-module connectivity (Pi). Grey dots represent peripheral nodes, and blue dots represent connectors. (**B**) Box plots of node Pi values (left) and Zi values (right) under different treatments. The Kruskal-Wallis test showed that the Pi values were not significantly different among groups (*P* = 0.14), whereas the Zi values differed significantly (*P* = 0.048)

As shown in Fig. 7A, the fungal network nodes were classified into peripheral nodes and connectors. Nodes across all treatments were predominantly peripheral, with only a minimal number of connectors present. While DR and MT did not significantly alter the overall distribution pattern of nodes, the number of connectors slightly increased under MT treatment. According to the node connectivity distribution presented in Fig. 7B, the Pi values for the CK, DR, and MT groups were all relatively low, with no significant differences observed among the groups (*P* = 0.14). In contrast, the Zi value for CK was significantly higher than those for DR and MT (*P* = 0.048), indicating that both DR and MT treatments weakened the within-module connectivity strength of fungal nodes.

**Fig. 8:**
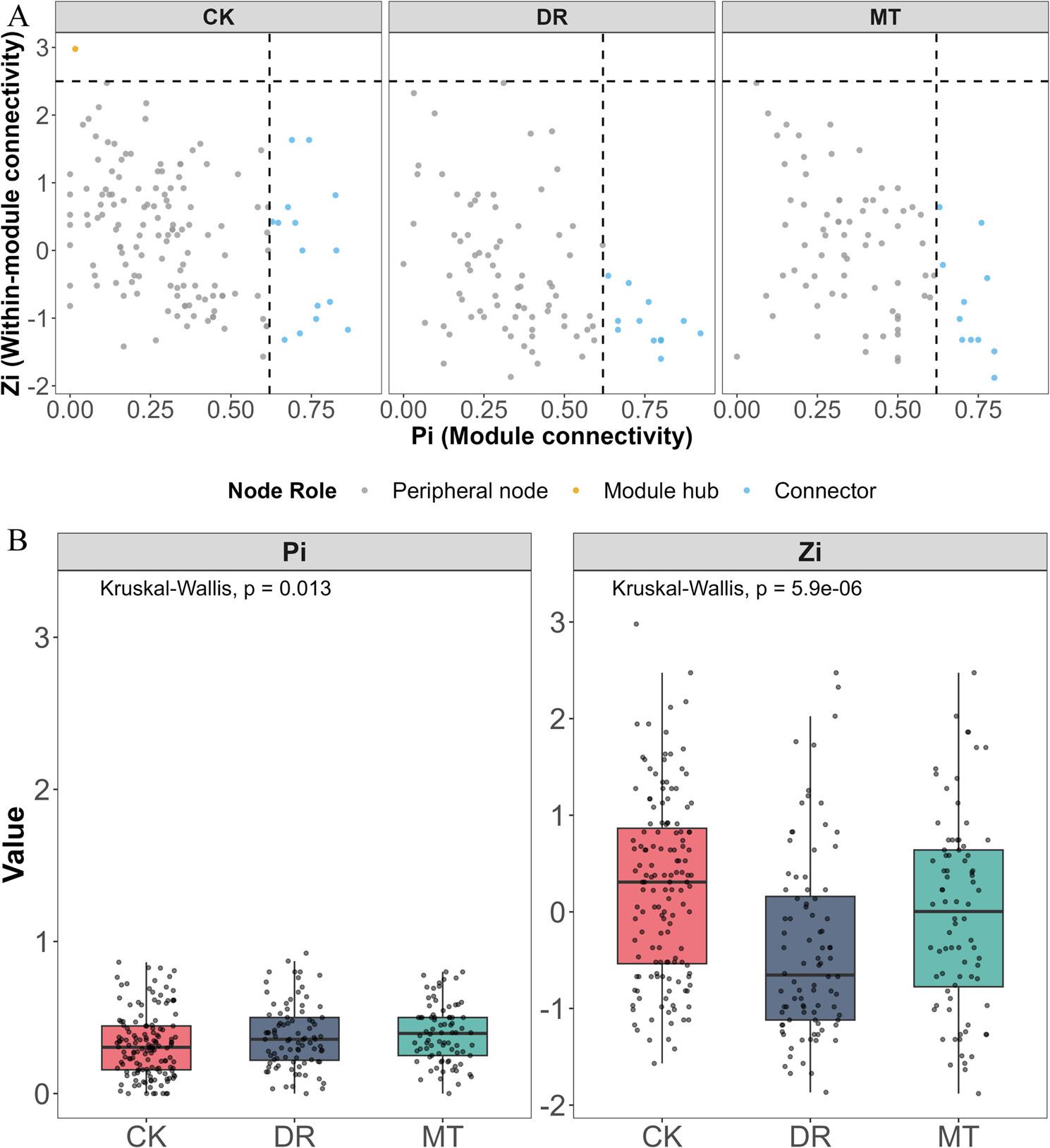
Zi-Pi analysis of bacterial network nodes in the rhizosphere soil of *Bougainvillea*. **A**. Scatter plot of node roles based on within-module connectivity (Zi) and between-module connectivity (Pi). Grey dots represent peripheral nodes, yellow dots represent Moduule hub, and blue dots represent connectors. **B**. Box plots of node Pi values (left) and Zi values (right) under different treatments. The Kruskal-Wallis test showed that the Pi values were not significantly different among groups (*P* = 0.14), whereas the Zi values differed significantly (*P* = 0.048)

Figure 8. Zi-Pi analysis of bacterial network nodes in the rhizosphere soil of *Bougainvillea*. (A) Scatter plot of node roles based on within-module connectivity (Zi) and between-module connectivity (Pi). Grey dots represent peripheral nodes, yellow dots represent Moduule hub, and blue dots represent connectors. (B) Box plots of node Pi values (left) and Zi values (right) under different treatments. The Kruskal-Wallis test showed that the Pi values were not significantly different among groups (*P* = 0.14), whereas the Zi values differed significantly (*P* = 0.048). Similarly, the bacterial network was dominated by peripheral nodes, but also contained a certain number of module hubs and connectors. Both DR and MT treatments reduced the number of connectors, indicating a weakening of inter-module connectivity in the bacterial network, (Fig. 8A). The Pi values for DR and MT were significantly higher than those for CK, suggesting that despite the decrease in the number of connectors, the cross-module connectivity of the remaining nodes was significantly enhanced. Compared to CK, DR significantly decreased the Zi value, while the Zi value in the MT group showed a recovery compared to DR, (Fig. 8B). This indicates that drought stress weakened the within-module connectivity strength, whereas melatonin partially alleviated this weakening effect and enhanced the connection capability of nodes within their modules.

### Redundancy analysis of physiological indicators of *Bougainvillea* plants and rhizosphere soil microbial communities

**Fig. 9:**
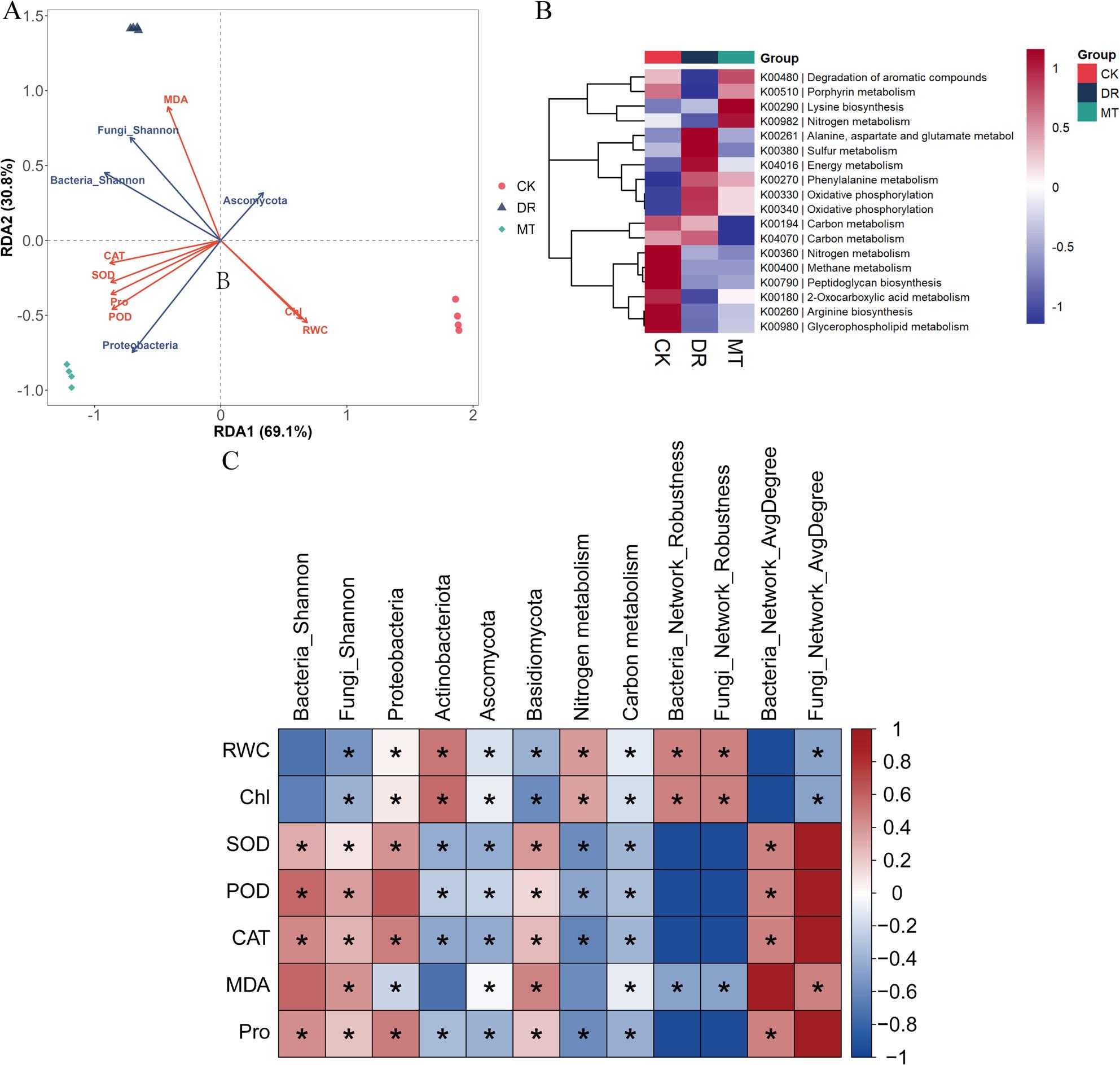
Comprehensive analysis of the association between physiological indicators of *Bougainvillea* and rhizosphere microbial characteristics with functional prediction of bacteria. **A**. RDA of physiological indicators and rhizosphere microbial metrics; **B**. Clustering heatmap of KEGG-based functional prediction for the bacterial community; **C**. Heatmap of Spearman correlation analysis between physiological indicators and rhizosphere microbial metrics (*P < 0.05, **P < 0.01)

RDA was performed to investigate the associations between physiological indicators of *Bougainvillea* plants and rhizosphere soil microbial communities. The first two RDA axes accounted for 69.1% (RDA1) and 30.8% (RDA2) of the total variance, respectively. CK samples were positively correlated with indicators such as RWC (relative water content) and Chl (chlorophyll content); the arrows of RWC and Chl were closely aligned, indicating a positive correlation between them. DR samples showed significant positive correlations with MDA, Fungi_Shannon (fungal Shannon index), and Ascomycota. MT samples were significantly positively correlated with Proteobacteria, SOD, POD, CAT, and Pro. The arrows of SOD, POD, CAT, and Pro pointed in similar directions, suggesting strong positive correlations among these indicators (Fig. 9A).

### Functional prediction of the rhizosphere soil bacterial community in *Bougainvillea* under drought stress following exogenous melatonin application

To gain deeper insights into the regulatory effects of exogenous melatonin on the functional potential of the rhizosphere soil bacterial community in *Bougainvillea* under drought stress, functional differences were analyzed using PICRUSt2 based on KEGG functional annotation and abundance clustering heatmaps. Distinct functional abundance profiles were observed among the different treatments, with CK, DR, and MT samples each clustering separately. This indicates that both drought stress and melatonin application significantly altered the functional spectrum of the bacterial community. In the CK group, the abundances of functional pathways such as arginine biosynthesis (K00260), 2-oxocarboxylic acid metabolism (K00180), peptidoglycan biosynthesis (K00790), and methane metabolism (K00400) were significantly increased. In the DR group, pathways related to energy metabolism, including alanine, aspartate and glutamate metabolism (K00261) and oxidative phosphorylation (K00330, K00340), showed significantly elevated abundances, while the abundances of pathways such as carbon metabolism (K00194) and degradation of aromatic compounds (K00480) were significantly reduced. In the MT group, the abundances of pathways including lysine biosynthesis (K00290), nitrogen metabolism (K00982), and energy metabolism (K04016) were significantly increased. Concurrently, the abundances of pathways such as carbon metabolism (K04070) and glycerophospholipid metabolism (K00980) were restored to levels comparable to those in the CK group (Fig. 9B).

### Correlation analysis between physiological indicators of *Bougainvillea* plants and rhizosphere soil microbial communities

Spearman correlation analysis was performed to investigate the relationships between aboveground physiological indicators of *Bougainvillea* and the rhizosphere soil microbial community as well as network characteristics. The results revealed that RWC showed significant positive correlations with Actinobacteriota, Ascomycota, Nitrogen metabolism (K00982), Carbon metabolism (K00194), and fungal network robustness (*P* < 0.05), while exhibiting significant negative correlations with Proteobacteria, Basidiomycota, and bacterial network robustness (*P* < 0.05). The correlation pattern of Chl was highly consistent with that of RWC. SOD, POD, and CAT exhibited significant positive correlations with Proteobacteria, bacterial network robustness, bacterial network average degree (Bacteria_Network_AvgDegree), and fungal network average degree (Fungi_Network_AvgDegree) (*P* < 0.05), while showing significant negative correlations with Actinobacteriota and Ascomycota (*P* < 0.05). Pro exhibited significant positive correlations with Proteobacteria, bacterial network robustness, bacterial network average degree, and fungal network average degree, while showing significant negative correlations with Actinobacteriota and Ascomycota. MDA showed a significant positive correlation with Proteobacteria (*P* < 0.05), and significant negative correlations with Actinobacteriota, Ascomycota, carbon metabolism (K00194), and fungal network robustness (Fig. 9C).

## Discussion

Drought stress often causes excessive accumulation of reactive oxygen species (ROS) in plants, resulting in oxidative damage to membrane systems and other cellular components. Therefore, enhancement of antioxidant defenses, including both ROS-scavenging compounds and antioxidant enzyme activities, is essential for maintaining redox homeostasis under water deficit conditions [38]. Exogenous melatonin has been widely reported to improve plant drought tolerance through regulation of antioxidant systems [39]. In this study, we found that under drought stress, melatonin significantly enhanced the activities of SOD, POD, and CAT in *Bougainvillea* leaves, which is consistent with our Hypothesis 1. This indicates that exogenous melatonin strengthens the antioxidant defense capacity of *Bougainvillea*, effectively scavenges ROS, stabilizes cellular structure, and thereby alleviates damage caused by drought stress. From a mechanistic perspective, melatonin is increasingly recognized as an important regulator of redox homeostasis in plants. ROS and reactive nitrogen species (RNS) are not only harmful by-products of stress but also key signaling molecules involved in stress perception and adaptation [40]. Melatonin may contribute to drought tolerance by modulating ROS/RNS balance, maintaining cellular redox status, and stabilizing stress-responsive metabolic processes [40–44]. In addition, melatonin has been reported to interact with phytohormone-related pathways, including ABA-associated and other stress-responsive regulatory pathways, thereby influencing stomatal behavior, osmotic regulation, and stress acclimation [45–47]. Emerging evidence also suggests potential interactions between melatonin and auxin-related pathways, which may affect root development, adaptive growth, and plant–microbiome interactions under stress conditions [45, 48]. Although these molecular signaling pathways were not directly examined in the present study, they provide a plausible mechanistic basis for the physiological improvements observed here.

The interaction between plants and rhizosphere microorganisms plays a central regulatory role in plant responses to both biotic and abiotic stresses [49]. Increasing evidence suggests that exogenous melatonin can participate in the reprogramming of the rhizosphere microbial community under stress conditions and acts as a key effector molecule in regulating the rhizosphere microecology [28]. For example, under drought stress, melatonin increased the relative abundance of bacteria associated with carbohydrate and carboxylate degradation pathways in the rhizosphere of barley [20]. Similarly, Zhang et al. [28] demonstrated that melatonin significantly reshapes the microbial community structure in the root zone of *Diospyros lotus* and induces the recruitment of potentially beneficial microorganisms, thereby enhancing its drought tolerance. Consistent with these findings, our results indicate that drought stress significantly altered the rhizosphere bacterial community structure, melatonin further adjusted the relative abundance of dominant microbial taxa (Figs. 3C and 4C). Melatonin did not significantly affect the α-diversity of fungal or bacterial communities(Fig.S1, Fig.S2), but selectively reshaped community composition. In particular, Proteobacteria remained the dominant bacterial phylum, and its relative abundance was higher under MT treatment than under CK and DR treatments (Fig. 4C). As members of Proteobacteria are often associated with nutrient turnover and plant stress adaptation, their enrichment may be functionally relevant [27], At the genus level, *Sphingomonas* showed a similar enrichment trend under DR and MT treatments (Fig. S3B), and previous studies have shown that this genus can improve plant growth and drought resistance by enhancing nutrient uptake and regulating root-associated microbial interactions [50]. Functional prediction further indicated that melatonin alleviated drought-induced inhibition of carbon metabolism pathways (Fig. 7B), which is consistent with the enrichment of these potentially beneficial bacterial taxa. Furthermore, in the fungal community, exogenous melatonin reduced the relative abundance of Ascomycota but increased the abundance of *Chloridium* under drought stress (Fig. S3A), suggesting a selective effect on fungal community assembly [51]. We speculate that melatonin-induced changes in plant physiological status, including redox balance, osmotic adjustment, and carbon metabolism, may alter root exudate composition and thereby reshape the rhizosphere microenvironment, favoring the recruitment of microorganisms involved in nutrient cycling and stress mitigation [52]. However, as root exudates, rhizosphere metabolites, and microbial functions were not directly verified in this study, these taxa should be regarded as candidate beneficial microorganisms rather than confirmed causal drivers. Future studies should further investigate root exudate dynamics, soil nutrient status, and functional validation of specific microbial taxa to clarify the mechanisms underlying melatonin-enhanced drought tolerance in *Bougainvillea*.

Microbial network analysis provides comprehensive insights into the dynamics of microbial communities [53]. Previous studies have clearly demonstrated that drought stress, as a typical abiotic stress, significantly disrupts the structure of rhizosphere microbial networks and reduces their complexity and stability [54, 55]. Specifically, Wang et al. [33] found that drought reduces the complexity of grassland microbial networks, and Peng et al. [56] further confirmed that drought stress simplifies microbial networks in both monoculture and intercropping systems. In contrast, our study shows that the DR treatment increased network edges, nodes (Fig. 5), as well as positive links, negative links, and average degree (Table 1), indicating a more complex microbial network under drought stress. This suggests that drought may promote rhizosphere microbial interactions, intensifying both cooperation and competition among microorganisms and resulting in a more intricate interaction network [57]. While increased network complexity generally enhances stability [35], our findings align with most existing studies in demonstrating that drought stress significantly reduces the stability of the rhizosphere microbial network in *Bougainvillea*. Specifically, this is reflected in decreased network robustness, increased vulnerability (Fig. 7), reduced within-module connectivity of fungal and bacterial nodes (Fig. 8), and marked disruption of core microbial interactions [58–60]. Consequently, the increased network complexity observed under drought may reflect instability, rendering the microbial community more susceptible to disturbance. Notably, in this study, exogenous melatonin treatment effectively reversed these drought-induced adverse effects and significantly enhanced the stability of the rhizosphere microbial network in *Bougainvillea*. The specific regulatory effects were manifested through optimized microbial network architecture, strengthened connectivity among keystone nodes, improved robustness, and reduced vulnerability, thereby stabilizing microbial interactions. These results are consistent with our Hypothesis 2 and provide evidence for melatonin-mediated drought resistance regulation in *Bougainvillea*.

Substantial studies have confirmed that rhizosphere microorganisms can improve plant growth performance and stress resistance under adverse conditions by regulating pathways such as nutrient cycling and hormone metabolism [61, 62]. Under drought stress, morphological and metabolic changes in plants significantly alter the composition and structure of the rhizosphere microbial community [63–65], while the host plant can also recruit beneficial microorganisms through metabolic signals in a tailored manner to adapt to the stressful environment [66]. Previous studies have demonstrated that exogenous melatonin can enhance plant resistance to abiotic stress by modulating the structure of the rhizosphere microbial community and recruiting beneficial functional microbiota [20, 27]. The findings of this study further reveal that exogenous melatonin mediates the aboveground-belowground synergistic drought resistance regulation in *Bougainvillea*. Correlation heatmaps demonstrated that, following melatonin treatment, significant correlations were observed between aboveground drought resistance physiological indicators (such as photosynthetic efficiency, antioxidant enzyme systems, and osmotic adjustment substances) and rhizosphere microbial characteristics (e.g., the abundance of the dominant phylum Proteobacteria and community diversity). RDA further confirmed that rhizosphere microbial community characteristics exhibited high explanatory power for the variation in aboveground physiological indicators. Moreover, the MT group showed significantly positive correlations with indicators such as Proteobacteria, SOD, POD, CAT, and Pro. In summary, exogenous melatonin can synergistically improve aboveground physiological drought resistance traits by modulating the structure and interactions of the *Bougainvillea* rhizosphere microbial community under drought stress, thereby establishing an aboveground-belowground synergistic drought resistance mechanism. These findings directly support Scientific Hypothesis 3 and closely align with the conclusions of Ye et al. [20] in barley, which demonstrated that melatonin enhances drought resistance by activating the antioxidant enzyme system and remodeling the rhizosphere microbial community.

### Limitations and future perspectives

Although this study showed that exogenous melatonin improved drought tolerance in *Bougainvillea* and was associated with selective shifts in the rhizosphere microbiome, several limitations remain. First, the current results are largely correlative, because the relationships between plant traits and microbial community changes were inferred mainly from community profiling, RDA, and network analyses. Thus, the differentially enriched taxa identified here should be considered candidate beneficial microorganisms rather than confirmed causal agents. Future studies should include microbial isolation, suspension inoculation, and sterilized soil experiments to test causality and to distinguish the relative contributions of plant-driven and microbe-driven mechanisms [67, 68]. Second, although melatonin appeared to simultaneously regulate antioxidant capacity and rhizosphere microbial structure, the mechanistic link between these processes was not directly examined. Root exudate composition, rhizosphere metabolites, and signaling molecules should therefore be analyzed in future work to clarify how melatonin-mediated physiological regulation influences microbial recruitment under drought [69, 70]. Third, root anatomical traits are closely linked to drought adaptation and resource acquisition, and should therefore be considered in future studies [9, 71–74]. In addition, because the present experiment used a single melatonin concentration under controlled drought conditions, further studies are needed to optimize melatonin application strategies under prolonged drought by testing different concentrations, frequencies, and application times [10, 75]. Finally, field trials across different soil types and climatic conditions will be necessary to evaluate the stability and practical applicability of the physiological and microbiome responses observed here, given that root-associated microbial communities are strongly influenced by host selection, soil properties, and environmental conditions [76, 77].

## Conclusion

**Fig. 10:**
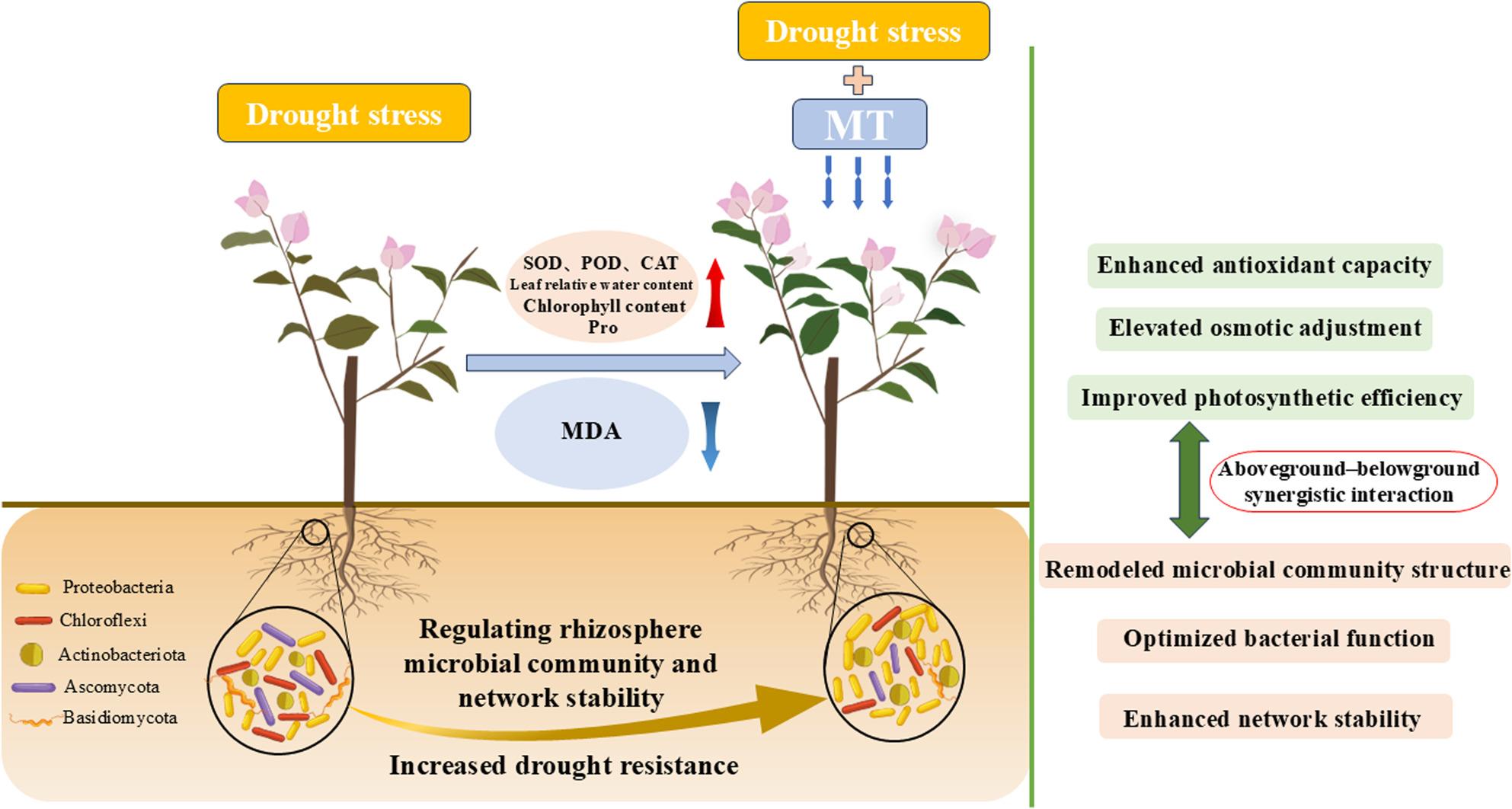
Conceptual model diagram of Melatonin-mediated alleviation of drought stress via modulation of physio-biochemical and rhizosphere microbial community structure in *Bougainvillea*

In this study, we systematically investigated the regulatory effects of exogenous melatonin on the growth physiology and rhizosphere microbiota of *Bougainvillea* under drought stress. Our findings demonstrate that exogenous melatonin effectively alleviates drought-induced damage by enhancing the plant’s antioxidant capacity, reducing membrane lipid peroxidation, and maintaining cellular osmotic balance. Simultaneously, exogenous melatonin was shown to reshape the diversity and composition of the rhizosphere microbial community under drought stress, while optimizing the structural properties of the microbial co-occurrence network. Comprehensive analysis revealed that melatonin mediates an aboveground–belowground coordinated drought resistance mechanism by synergistically regulating the drought-resistant physiology of the aerial parts of the plant and the structure and network stability of the rhizosphere microbial community, thereby enhancing the drought tolerance of *Bougainvillea*. This study clarifies the important role of melatonin in improving the drought resistance of the woody ornamental plant *Bougainvillea* and highlights the critical significance of rhizosphere microorganisms in the process of melatonin-mediated drought stress alleviation. The findings provide a theoretical basis and technical support for drought-resistant cultivation of Bougainvillea and rhizosphere microecological management. (Fig. 10).

## Supplementary Information

Supplementary Material 1.

Supplementary Material 2.

## Data Availability

The data generated or analyzed are provided within the manuscript and its additional file. The datasets analyzed during the current study are available from the corresponding author upon reasonable request.
