# Supplementary material for: Melatonin-mediated dual regulation of physiological-biochemical traits and rhizosphere microbial communities alleviates drought stress in Bougainvillea
Source: BMC Plant Biol. 2026 Jun 6;26:1352. doi: 10.1186/s12870-026-09179-1 (PMC13459286; doi:10.1186/s12870-026-09179-1)
Supplement: Supplementary file 1 [file 12870_2026_9179_MOESM1_ESM.docx]

Fig. S1. Effects of exogenous melatonin on the rhizosphere soil fungal alpha diversity of *bougainvillea* under drought stress. A. Ace index; B. Chao index; C. Shannon index; D. Simpson index.

Fig. S2 Effects of exogenous melatonin on the rhizosphere soil bacterial alpha diversity of *bougainvillea* under drought stress. A. Ace index; B. Chao index; C. Shannon index; D. Simpson index.

Fig. S3 Effect of exogenous melatonin on the composition of soil microbial communities in the rhizosphere of *bougainvillea* under drought stress. A. The relative abundance of fungi at the genus level; B. The relative abundance of bacteria at the genus level.
