# Supplementary material for: Melatonin-mediated dual regulation of physiological-biochemical traits and rhizosphere microbial communities alleviates drought stress in Bougainvillea
Source: BMC Plant Biol. 2026 Jun 6;26:1352. doi: 10.1186/s12870-026-09179-1 (PMC13459286; doi:10.1186/s12870-026-09179-1)
Supplement: Supplementary file 2 [file 12870_2026_9179_MOESM2_ESM.docx]

Determination of Physiological Parameters

For enzyme activity analysis, plant tissue (0.1 g) was ground in liquid nitrogen and homogenized with 2 mL phosphate buffer (pH 7.8). After centrifugation (10,000 rpm, 25 min, 4℃), the supernatant was collected as crude enzyme extract and stored at 4℃ for SOD, POD and CAT activity measurements. All assays included three technical replicates with data reported as means.

The NBT photoreduction method served as the basis for quantifying superoxide dismutase (SOD) activity. The sample tubes received 50 µL of enzyme extract, whereas the control tubes were supplemented with an equal volume of phosphate buffer. All analyses were carried out with three technical replicates. Each tube received a reaction mixture prepared by sequentially adding 1.5 mL phosphate buffer, 0.3 mL of 130 mmol·L⁻¹ methionine (Met), 0.3 mL of 750 μmol·L⁻¹ NBT, 0.3 mL of 100 μmol·L⁻¹ EDTA-Na₂, 0.3 mL of 20 μmol·L⁻¹ riboflavin (RFN), and 0.25 mL distilled water. Following thorough mixing, the tubes were illuminated at 4000 lx for 30 min. Absorbance at 560 nm was determined using a microplate reader. SOD activity was derived from the following equation:

SOD activity (U·g⁻¹)=[(Aₖ-Aₛ)×Vₜ]/(0.5×Aₖ×W×Vₛ)

where:Aₖ=absorbance of the control tube at 560 nm; Aₛ=absorbance of the sample tube at 560 nm; Vₜ=total volume of enzyme extract (mL); W=fresh tissue weight (g); Vₛ=volume of enzyme extract used in reaction (mL)

Peroxidase (POD) Activity Determination by Guaiacol Oxidation Method. The POD reaction solution was prepared by first adding 56 μL of guaiacol to 100 mL of phosphate buffer (pH 6.0). After complete dissolution using a heated stirrer, the solution was cooled to room temperature and supplemented with 38 μL of 30% H₂O₂. For the assay, each tube received 20 μL of enzyme extract combined with 3 mL of the prepared reaction solution. All samples were analyzed in three independent replicates. A microplate reader was employed to track the rising absorbance at 470 nm through continuous measurement at 1-minute intervals for the duration of three minutes. POD activity was calculated based on the following equation:

POD activity [U·(min·g)⁻¹]=(ΔA₄₇₀×Vₜ)/(W×Vₛ×0.01×t)

where:ΔA₄₇₀=change in absorbance at 470 nm during the reaction period; Vₜ=total volume of enzyme extract (mL); W=fresh tissue weight (g); Vₛ=volume of enzyme extract used in assay (mL); t=total reaction time (min)

Catalase (CAT) activity was determined by reacting 100 μL of enzyme extract with 0.5 mL of 0.1 mol·L⁻¹ H₂O₂ and 2 mL phosphate buffer (0.1 mol·L⁻¹, pH 7.0). The decrease in absorbance at 240 nm was monitored every minute for 3 minutes using a microplate reader. Enzyme activity was determined using the following calculation:

CAT activity [U·(min·g)⁻¹]=(ΔA₂₄₀×Vₜ)/(W×Vₛ×0.1×t)

where: ΔA₂₄₀=decrease in absorbance at 240 nm per minute; Vₜ=total volume of enzyme extract (mL); W=fresh tissue weight (g); Vₛ=volume of enzyme extract used in reaction (mL); t=reaction duration (min)

Quantification of Malondialdehyde (MDA) Using Thiobarbituric Acid Reaction. Plant tissue (0.1 g) was cryogenically pulverized in a liquid nitrogen-chilled mortar. Subsequent homogenization was performed with 2 mL of 5% trichloroacetic acid (TCA), followed by transfer of the homogenate to a 2 mL centrifuge tube. After centrifugation at 10,000 rpm for 20 min (4°C), the supernatant was collected for analysis. For the assay, 1 mL of supernatant was combined with 1 mL of 0.6% thiobarbituric acid (TBA), while a control reaction contained 1 mL of 5% TCA with 1 mL TBA. After a 15-min boiling water bath incubation and subsequent cooling, the sealed mixtures were centrifuged at 5,000 rpm for 10 min (room temperature). The cleared supernatant was then collected for absorbance measurement at 450, 532, and 600 nm. MDA concentration was determined using the following equation:

MDA content (mmol·g⁻¹)={[6.45×(A₅₃₂-A₆₀₀)-0.56×A₄₅₀]×Vₜ×V}/(W×Vₛ)

where: A₄₅₀, A₅₃₂, A₆₀₀=absorbance values at respective wavelengths; Vₜ=total volume of test solution (mL); V=total volume of extraction solution (mL); W=fresh tissue weight (g); Vₛ=volume of extraction solution used in assay (mL)

Proline(Pro) concentration was quantified with a commercial assay kit (Beijing Hezi Biotechnology Co., Ltd.). Following kit instructions, a standard curve was prepared prior to sample analysis. After homogenizing 0.1 g fresh tissue in 1 mL extraction solution and heating in a boiling water bath for 10 min (with 2-3 vortex mixing cycles), the cooled homogenate was centrifuged at 10,000 rpm for 10 min at room temperature. The clear supernatant was used for proline determination.Reaction mixtures were prepared as follows:

Test: 200 μL supernatant+200 μL glacial acetic acid+200 μL chromogenic solution

Standard: 200 μL standard solution+200 μL glacial acetic acid+200 μL chromogenic solution

Blank: 200 μL distilled water+200 μL glacial acetic acid+200 μL chromogenic solution

After thorough mixing, all tubes were heated at 100°C for 30 min with periodic mixing, cooled to room temperature, and supplemented with 400 μL toluene. Following vigorous vortexing for 30 sec and phase separation, the upper organic layer was collected for absorbance measurement at 520 nm. Proline content was calculated as:

Proline content (μg·g⁻¹)=(C×V)/W

where: C=proline concentration (μg·mL⁻¹) determined from standard curve; V=total volume of test sample (mL); W=fresh tissue weight (g)

The standard curve was established as y=0.0317x-0.0351 (R²=0.9955), with sample concentrations derived by inserting corrected absorbance values into this equation.
